# Dental Nomenclature

**Published:** 1883-10

**Authors:** 


### THE DENTAL REGISTER.

VOL. XXXVIII.] OCTOBER, 1883. [No. 10.

### COMMUNICATIONS.

### Dental Nomenclature.

[Extract from the report on this subject read before the American Dental Association, August 8, 1883.]

In medical literature a large proportion of the distinctive names are taken, changed but little if any, from other languages, and chiefly from the Greek and Latin. The propriety of anglicising these names is a question about which there is some diversity of opinion.

The rendering more plain and understandable these distinctive names and phrases would seem to be very desirable; in as much as a large majority of those engaged in the study of the branches in which they so greatly abound, have but little or no knowledge of any other language than English. Often times the student will struggle with names and phrases without a knowledge of their meaning for months and even years. Everything that can be as well designated by the pure English word or phrase should not be named by words of another language and especially is this true since a large proportion of those who study the professions are not familiar with those languages and especially the Greek and Latin. Usually the Greek and Latin names used in descriptive anatomy and pathology are not more expressive nor better in any sense than well chosen English words and phrases. A large share of the labor in mastering these branches consists in becoming familiar with the nomenclature. The growth of language is slow, and change cannot be rapidly introduced. In the use of language for conveying thought, that which is best adapted should be employed using only those words and terms that will most clearly and fully designate the thought, object, or action. Many names are used upon analogy or association, that will hardly stand the test of close criticism. To some of these reference will be made as we proceed. It is said that in the development of art and science new words and phrases are a necessity.

But in introducing these caution should be exercised, every new word, name, or phrase introduced should be made clear and comprehensible to every reader before it is generally employed. Its origin and significance should be such as to commend it to the judgment of every one of general culture. In the art and science of the dental profession there are many distinctive names and phrases. For the most part perhaps these are well chosen, and as expressive as it is possible in the present state of our language. There is, however, quite a number of nominations of very questionable propriety. To a few of these we now propose to call attention. The different teeth have different names, some of these sufficiently distinctive, as for example, the incisors, the molars, the bicuspids, the cuspids, etc.

To some of these, however, names are given which are of doubtful propriety, for example, the cuspid teeth both superior and inferior are often called canine teeth. This simply because they proximate the form of the dog's tooth; this is certainly a very faulty basis for a distinctive name, in as much as many other animals have teeth of a very similar form; indeed, quite as pointed as those of the dog. The word cuspid means a point and when applied to a tooth means a pointed tooth. No other word can better designate this tooth. Plurality of names tends to confusion. These teeth are also sometimes called eye-teeth as applied to the superior and stomach teeth as applied to the inferior. These appellations are wholly unwarranted by the forms of the teeth or by any relation they bear to the parts referred to in these names.

The first permanent molar is often called the "six year molar," and sometimes the "six year old molar," the latter being a crude expression that no one of just taste would tolerate for a moment. The ground of this nomination is the fact that this tooth is usually erupted when its possessor is about six years of age.

The inappropriateness of this name is apparent from the fact that the tooth may appear at any time from five and a half to seven years of age and in some instances even later. The name as used would seem to indicate the age of the tooth rather than that of the possessor. There is no more propriety in thus designating these teeth than in calling the second molar the twelve year molar or the third molar the eighteen year molar, or of naming the bicuspids according to the years of their eruption. The appellation first permanent molar is clear and distinct and cannot apply to any other tooth ; let this then be the name by which this tooth shall be designated. The third molar is known by several names. Some think it entitled to the Latin name and hence call it *dentes-sapientiæ*, and others are content with the same name in English and hence call it the wisdom tooth. The better and more distinctive name, however, is the third molar ; this is clear and understandable by all, and is quite sufficient for its designation. The multiplicity of names always tends to confusion.

That part of the teeth resting in the alveolus and by which the attachment is made is denominated the root and the fang.

The latter name was in far more common use some years ago than now ; one of these names is quite sufficient to designate this part of the tooth, and the word root is certainly more appropriate than the word fang. No one could be mistaken by this application of this word, while fang is more appropriately applied to the sharp pointed body that protrudes from a socket or base. Nothing in any respect is gained by the use of these two words to designate that part of the tooth by which it is attached to the jaw. Let the word root then be adopted for this designation to the exclusion of the word fang.

Having heard the anterior root of a lower molar defined as the mesial root and not being able to appreciate the sense of such a definition, it is proposed here to bring before the Association the terms mesial and distal in order to get a true definition and the proper use of these terms.

Mesial, Greek *mesos* middle; anatomical definition "medical lexicons," an imaginary plane dividing the head, neck, and trunk into similar halves, towards right or left, every aspect towards this plane being mesial, and every aspect towards right or left being lateral, every lateral aspect being either dextral or sinistral. In the human subject the teeth are arranged around the margins of the jaws in a parabolic curve or proximating it, and special names having been applied to various surfaces on this account with reference to front, back, or sides in order to indicate the position occupied by the teeth and their relation to definite terms universally made use of by anatomists, to-wit: median anterior, and posterior. It is, therefore, correct to designate the proximating surfaces of the central incisors as the mesial surface, because the position occupied by them fulfills the conception we have, that if the median line passes between them, that the surface looking towards this line is the mesial surface. The labial surface representing the anterior, and the lingual, the posterior surface of a central incisor. But as before stated the maxillary forming a parabolic curve, it stands to reason when this term is applied to a bicuspid that the position of surfaces change entirely, that is if the same nomenclature is employed the surface that was properly designated mesial in the incisors becomes an anterior surface in the bicuspid in either the upper or lower maxillary, and what was denominated anterior and posterior in relation to the median line must now either be defined as labial and lingual, or, if you will, carrying out the meaning of the word mesial, designate the lingual surface as the mesial, for it is the only surface that looks towards the median line.

Dentine—the word dentine has been long in use, and the usual one designating that part of the tooth constituting its body which is found inclosed by the enamel and the cement. Perhaps no better term than this can be found. The word *dentos* has been suggested as a substitute for the word dentine, but why the change should be made we do not readily see.

The word dentos may be appropriately used as a name for the bony covering of the dentine of the root of the tooth. This material is usually denominated the cement or cementum; both meaning the same thing, cementum being the Latin form of the word, cement the English. This outer portion of the root of the tooth partakes much more nearly in structural character that of bone than dentine and may with propriety be demoninated dentos. This word would be far better restricted in its application to this part of the tooth. Dr. Chase, of St. Louis, some years ago suggested the use of this name. However, he may have intended, it has been used by many as designating all the hard structures of the teeth, and by some the dentine merely. This word is an illustration of certain cases in which it seems better to use the word of another language. The cases in which this is necessary are few and the number should be lessened whenever practicable.

Tooth pulp—This soft, highly organized body occupying the central portion of every tooth is frequently called the nerve of the tooth. Many of our best writers, from custom or habit rather than from a just appreciation have used this name. It has perhaps received this nomination because of its extreme sensitivenss when exposed to external contact.

The inappropriateness of this name is apparent when we remember that only a very small part of this body constitutes the actual nerve tissue. It is made up of a small portion of membranous and fibrous tissue, of a large amount of fluid or s mi-fluid, veins and arteries, together with a small amount of nerve tissue. There is no more propriety in denominating this body a nerve than in so designating any other highly sensitive and finely organized tissue or part of the body. The eye, for instance, as a whole may be as properly designated a nerve. The word nerve as applied to this tissue is to the student at least misleading. This structure has sometimes very inappropriately been called the lining membrane of the tooth, and hence some writers of former years spoke of destroying or removing the lining membrane, evidently meaning the pulp of the tooth. The word pulp, or tooth pulp, is sufficiently distinctive and understandable and could not be mistaken for anything else and would convey more nearly the idea of the structural character of the tissue. It has been suggested that the use of this name might also indicate that pulp mass, from which the tooth grows. This is readily distinguishable, however, by the word embryo. Is it not proper and desirable, therefore, to designate this soft tissue as the pulp rather than the nerve?

Periosteum—This is the usual name given to that thin layer of soft tissue immediately surrounding the root of every tooth. Some have been disposed to use the word peridontium. For such a change we really perceive no valid reason. This tissue of course surrounds the dentine but is not in contact with it, the cement or dentos intervening, which in structure is more nearly allied to bone than the dentine and it is in actual contact with the former and not with the latter. If the desire is to be very definite and indicate that this structure is about the teeth rather than some other bone let the word dental be adopted. The phrase dental-periosteum is as easily spoken and really more distinctive and more euphonious than the word peridontium. When we speak of an inflamed condition of this tissue in conformity with this we would say periostitis instead of periodontitis.

Mechanical dentistry—This phrase has long been used to designate that part of dental science and art which has to do with the manufacture and insertion of artificial teeth and all that pertains to it. So far as the needs of mechanical and artistic skill is concerned this name may be sufficiently distinctive, but it is not sufficiently extended in its reach. Some may execute well in a mechanical aspect and yet signally fail in the production and application of substitutes for lost teeth and the restoration of adjacent parts. The name that will embrace both these ideas, namely, that of mechanism and of art, that shall enable the application of it to the parts here indicated is that which should be employed and in a search of some extent, no better word occurs for this name than dental-prosthesis. Some have been disposed to change the form of the phrase to prosthetic dentistry. The other is the better phrase, we think. This nomination is slowly but surely making its way in the literature of our profession. It will probably be used ere long to the exclusion of the phrase mechanical dentistry.

Operative dentistry—This name or phrase is usually employed, and properly so to designate operations upon the natural teeth for their preservation, or rescuing them from the ravages of disease. It means simply operations upon natural the teeth, and is sufficiently clear and distinctive. There has been a little disposition in some quarters to drop this name and embrace everything done in the mouth upon both the hard and soft tissues by the name of "Oral Surgery," this may with great propriety be applied to all operations upon the soft and hard parts in and immediately about the mouth, but the nature of the operations upon the teeth is so diverse from those performed upon the other parts of the mouth as to entitle it to a distinctive name. There is sometimes an effort made to have the name embrace too much, and this would seem to be a case of that kind.

Your committee in fulfillment of the task assigned them have deemed it proper to present these few illustrations of changes which may be appropriately made in the special nominations of our profession.

These are only a few to which reference may properly be made, but time would hardly permit a more extensive presentation of the subject, and we fully recognize that change of language is necessarily slow. It would be a waste of time to even suggest any very great or radical change with the hope that it would receive even brief consideration, but where there are manifest defects in our language and especially in its nominations and particularly those that are apparent to persons of general culture, and where names tend to confusion rather than to clear understanding, it behooves us to do what we may to remedy the defects. The great difficulty that lies in the way of changes, such as are here suggested, is found not so much in the question whether they are correct or not, as in the carelessness and want of attention by those who should be interested to make progress and secure the best form of distinctive literature.

